# Beyond Creatinine: Novel Renal Biomarkers at the Interface of Kidney Injury and Cardiovascular Risk

**DOI:** 10.3390/biomedicines14071525

**Published:** 2026-07-07

**Authors:** Maria-Daniela Tanasescu, Andrei-Mihnea Rosu, Alexandru Minca, Maria-Mihaela Grigorie, Delia Timofte, Dorin Ionescu

**Affiliations:** 1 Department of Semiology, Emergency University Hospital, Carol Davila University of Medicine and Pharmacy, 022328 Bucharest, Romania; maria.tanasescu@umfcd.ro (M.-D.T.); dorin.ionescu@umfcd.ro (D.I.); 2 Department of Cardiology, Prof. Dr. Agrippa Ionescu Emergency Hospital, 077015 Balotesti, Romania; 3 Department of Dentistry, Discipline of Endodontics, Faculty of Dentistry, Carol Davila University of Medicine and Pharmacy, 020021 Bucharest, Romania; maria.grigorie@umfcd.ro; 4 Department of Dialysis, Bucharest Emergency University Hospital, 050098 Bucharest, Romania; delia.timofte@gmail.com

**Keywords:** renal biomarkers, cardiovascular risk, cardiorenal syndrome, NGAL, KIM-1, cystatin C, pro-enkephalin, ST2, galectin-3, FGF-23

## Abstract

Chronic kidney disease, acute kidney injury and cardiorenal syndrome are major determinants of cardiovascular morbidity and mortality, yet conventional renal assessment based on serum creatinine, estimated glomerular filtration rate and urine output often fails to detect early structural injury or pathway-specific cardiorenal risk. This narrative review synthesized recent evidence on emerging renal and cardiorenal biomarkers with potential value for cardiovascular risk stratification beyond creatinine. Literature published between 2015 and April 2026 was reviewed, focusing on biomarkers of tubular injury, functional renal impairment, fibrosis/remodeling and mineral metabolism. NGAL and KIM-1 may detect tubular stress and proximal tubular injury before overt functional decline and have shown relevance in heart failure, acute coronary syndromes and post-cardiac surgery settings. Cystatin C and pro-enkephalin refine functional renal assessment and may improve prognostic classification when creatinine is confounded by frailty, muscle mass or acute hemodynamic changes. Soluble ST2 and galectin-3 reflect inflammation, fibrosis and cardiorenal remodeling, while FGF-23 links kidney dysfunction to cardiovascular risk through phosphate imbalance, vascular calcification and myocardial hypertrophy. Multi-biomarker panels may help identify dominant cardiorenal phenotypes and personalize monitoring intensity. However, routine implementation requires standardized assays, validated thresholds, cost-effectiveness data and prospective evidence that biomarker-guided management improves clinical outcomes.

## 1. Introduction

Chronic kidney disease (CKD) is now recognized as a major public health problem and a strong determinant of cardiovascular prognosis. The KDIGO 2024 guideline defines CKD as abnormalities of kidney structure or function persisting for at least three months, with implications for health, and classifies it according to cause, glomerular filtration rate (GFR) category, and albuminuria category [1]. This classification frames CKD as a continuum of risk, in which lower GFR and higher albuminuria identify patients at increased risk of both renal and extra-renal complications [1,2].

The burden of CKD extends well beyond kidney-related outcomes. Worldwide, all-stage CKD affects approximately 697.5 million people, with an estimated prevalence of 9.1%. It accounts for 1.2 million deaths and 35.8 million disability-adjusted life-years, while impaired kidney function contributes to a further 1.4 million cardiovascular disease-related deaths and 25.3 million cardiovascular disability-adjusted life-years. These figures place kidney dysfunction among the major contributors to global cardiovascular morbidity and mortality [3].

Lower estimated glomerular filtration rate (eGFR) and higher albuminuria are consistently associated with adverse renal and cardiovascular outcomes, including kidney failure, acute kidney injury (AKI), all-cause and cardiovascular mortality, hospitalization, coronary heart disease, stroke, heart failure (HF), atrial fibrillation, and peripheral artery disease [4]. Kidney dysfunction therefore cannot be interpreted only as a downstream consequence of cardiovascular disease (CVD) or hemodynamic instability. It may also contribute to cardiovascular risk through inflammation, endothelial dysfunction, vascular calcification, neurohormonal activation, and myocardial remodeling [3,4].

AKI adds another layer of cardiovascular risk and is often under-recognized after the acute event has resolved. In routine practice, AKI is diagnosed using serum creatinine (SCr), estimated GFR, and urine output. These functional markers have limited sensitivity and specificity, may require a baseline creatinine value that is not always available, and do not directly identify structural tubular injury. As a result, AKI may be misclassified or detected only after clinically relevant renal damage has occurred, particularly in patients with acute cardiovascular decompensation or critical illness [5].

The prognostic consequences of AKI often persist after discharge. AKI has been associated with higher risks of cardiovascular mortality, major cardiovascular events, HF, acute myocardial infarction, and stroke [6]. It is also linked to incident CKD, CKD progression, HF events, and all-cause mortality. Post-discharge assessment of kidney function recovery and proteinuria, particularly around three months after hospitalization, may add useful prognostic information and supports a structured approach to post-AKI risk stratification [7].

These observations are particularly relevant in cardiorenal syndrome (CRS), a condition in which acute or chronic dysfunction of the heart or kidneys induces dysfunction in the other organ. The American Heart Association scientific statement describes cardiorenal interactions as the result of hemodynamic mechanisms, including reduced cardiac output and venous congestion, together with neurohormonal activation, inflammation, oxidative stress, endothelial dysfunction, anemia, and mineral metabolism disturbances [8]. In this setting, SCr may fail to distinguish a functional change in filtration from structural tubular injury. Biomarkers that reflect tubular damage, altered filtration, fibrosis, remodeling, and mineral metabolism abnormalities may therefore refine early diagnosis and cardiovascular risk prediction. This narrative review summarizes the evidence supporting NGAL, KIM-1, cystatin C, pro-enkephalin, ST2, galectin-3, and FGF-23 as candidate tools for cardiorenal risk assessment beyond creatinine-based evaluation.

## 2. Narrative Review Methodology

This narrative review evaluates emerging renal and cardiorenal biomarkers in relation to early kidney injury detection, cardiorenal dysfunction, and cardiovascular risk stratification. The review focused on five biomarker domains: tubular injury markers, represented by neutrophil gelatinase-associated lipocalin (NGAL) and kidney injury molecule-1 (KIM-1); functional renal markers beyond SCr, including cystatin C and pro-enkephalin (PENK); fibrosis, inflammation, and remodeling markers, including soluble suppression of tumorigenicity-2 (sST2) and galectin-3; mineral metabolism-related biomarkers, particularly fibroblast growth factor-23 (FGF-23); and the potential clinical relevance of these markers in patients with CKD, AKI, HF, acute coronary syndromes, diabetes, hypertension, and cardiorenal syndrome.

A literature search was conducted in PubMed/MEDLINE, Scopus, and Web of Science for articles published from January 2015 to April 2026. The search combined Medical Subject Headings (MeSH) and free-text terms related to renal biomarkers, cardiorenal biomarkers, cardiovascular risk, cardiovascular outcomes, chronic kidney disease, acute kidney injury, cardiorenal syndrome, and the individual biomarkers discussed in this review. The main search terms included “renal biomarkers”, “kidney biomarkers”, “cardiorenal biomarkers”, “cardiovascular risk”, “cardiovascular disease”, “major adverse cardiovascular events”, “mortality”, “chronic kidney disease”, “acute kidney injury”, “cardiorenal syndrome”, “NGAL”, “neutrophil gelatinase-associated lipocalin”, “KIM-1”, “kidney injury molecule-1”, “cystatin C”, “pro-enkephalin”, “penKid”, “ST2”, “soluble ST2”, “galectin-3”, “fibrosis”, “cardiac remodeling”, “FGF-23”, “fibroblast growth factor-23”, “mineral metabolism”, and “vascular calcification”.

Reference lists of relevant original studies, systematic reviews, meta-analyses, consensus documents, and major cardiology and nephrology guidelines were also screened manually. Priority was given to studies examining associations between renal or cardiorenal biomarker elevation and clinically relevant cardiovascular outcomes, including HF hospitalization, acute coronary events, left ventricular hypertrophy, atherosclerotic burden, cardiorenal deterioration, cardiovascular mortality, and all-cause mortality.

Eligible sources included original research articles, observational cohort studies, randomized controlled trials, translational and mechanistic investigations, systematic reviews, meta-analyses, authoritative narrative reviews, clinical guidelines, and consensus statements from nephrology and cardiology societies. Articles published in English were prioritized. Case reports, editorials, letters, and conference abstracts were generally excluded, except when they offered mechanistic or conceptual information not adequately covered in full peer-reviewed articles.

Because this was a narrative review, no formal risk-of-bias assessment, quantitative quality scoring, or meta-analysis was performed. Study selection was guided by mechanistic relevance, clinical applicability, biological plausibility, and contribution to understanding the interface between renal injury, cardiorenal dysfunction, and cardiovascular risk. Particular attention was given to studies assessing biomarkers beyond conventional creatinine-based evaluation, especially those exploring their potential value for early diagnosis, prognostic stratification, phenotype identification, or individualized monitoring in high-risk cardiovascular and renal populations.

## 3. Pathophysiological Basis of the Cardiorenal Biomarker Axis

CRS refers to a bidirectional disorder in which acute or chronic dysfunction of the heart or kidneys contributes to dysfunction of the other organ. Its biology cannot be reduced to impaired perfusion alone. Hemodynamic stress, neurohormonal activation, inflammation, oxidative stress, endothelial dysfunction, fibrosis, and metabolic abnormalities interact across the heart–kidney axis and shape both renal and cardiovascular outcomes [8,9,10,11].

For clinical interpretation, CRS is commonly divided into five types according to the primary organ injury, the secondary organ dysfunction, and the acute or chronic time course of the process. This classification is useful because biomarker interpretation differs between acute cardiac decompensation with secondary kidney injury, chronic heart failure with progressive renal dysfunction, primary acute or chronic kidney disease with cardiovascular consequences, and systemic disorders that affect both organs simultaneously. The five CRS types are summarized in Table 1.

The classical model begins with renal hypoperfusion. In HF, reduced cardiac output may decrease renal arterial perfusion, activating the renin–angiotensin–aldosterone system (RAAS), the sympathetic nervous system, and vasopressin release. These responses help maintain circulatory volume in the short term, but persistent activation promotes sodium and water retention, vasoconstriction, increased preload, congestion, and further cardiac dysfunction [8,9]. Reduced forward flow, however, does not fully explain CRS. Renal filtration depends on the pressure gradient between arterial inflow and venous outflow; therefore, elevated central venous pressure and renal venous congestion may reduce intrarenal blood flow and impair glomerular filtration even when systemic blood pressure is preserved [8,9,10].

Venous congestion is an important contributor to kidney injury in CVD. Increased central venous and intra-abdominal pressures may reduce renal plasma flow, impair the filtration gradient, and contribute to oliguria and worsening renal function (WRF) in acute decompensated heart failure (ADHF) [10,11]. This helps explain why renal dysfunction may occur in both reduced and preserved ejection fraction heart failure, and why decongestion has clinical relevance beyond symptom relief [8,9].

Non-hemodynamic mechanisms further intensify the cardiorenal loop. Endothelial dysfunction reduces nitric oxide bioavailability and favors oxidative stress, inflammation, vascular permeability, extracellular matrix remodeling, and a prothrombotic state [1]. Inflammatory cytokines, including tumor necrosis factor-α, interleukin-1, and interleukin-6, may exert cardiodepressant effects, while sustained RAAS activation and reactive oxygen species contribute to tubular ischemia, myocardial remodeling, and progressive fibrosis [8,11]. This pathway diversity provides the biological rationale for biomarkers that reflect different components of cardiorenal injury, rather than relying only on SCr.

Conventional renal indices do not capture the full spectrum of kidney–heart injury. SCr rises after a substantial fall in GFR, may lag 48–72 h after AKI, and is influenced by age, sex, muscle mass, volume status, ethnicity, and drug exposure. It may therefore fail to separate functional renal changes from structural parenchymal injury. In CRS, biomarker interpretation is more informative when linked to dominant biological pathways, including filtration impairment, tubular injury, inflammation, oxidative stress, endothelial dysfunction, fibrosis, and mineral metabolism disturbance [12].

A second clinically useful distinction is between biomarkers that mainly inform acute cardiorenal injury and those that are more closely related to chronic remodeling, fibrosis, mineral metabolism disturbance, and long-term cardiovascular risk. This distinction is not absolute, because several biomarkers may be relevant in both acute and chronic settings. However, organizing biomarkers according to the dominant phase of injury may help clinicians interpret whether the signal mainly reflects acute tubular stress, functional renal deterioration, incomplete recovery after AKI, chronic cardiorenal remodeling, or CKD-related cardiovascular risk. This phase-based interpretation is summarized in Table 2.

Cystatin C and PENK reflect functional renal impairment beyond creatinine-based assessment. Cystatin C is less dependent on muscle mass than creatinine and has been associated with adverse outcomes in acute and chronic HF, although its concentration may still be affected by inflammation, thyroid disease, obesity, smoking, glucocorticoid exposure, and malignancy [12,13]. PENK A is inversely related to GFR and has been reported to predict AKI and adverse outcomes in acute HF, supporting its role as an emerging functional cardiorenal biomarker [12].

Tubular injury biomarkers add a different layer of information. NGAL is released by neutrophils, renal tubular epithelial cells, and cardiomyocytes in response to inflammation and injury. It may rise earlier than creatinine and has been linked to AKI prediction in cardiac surgery and acute HF [12,13]. KIM-1 reflects proximal tubular epithelial injury and may help identify structural tubular damage when changes in creatinine are delayed or clinically ambiguous [12]. Biomarkers of fibrosis and remodeling, including galectin-3 and sST2, have been associated with cardiac fibrosis, kidney disease progression, HF prognosis, and possibly diuretic resistance [12,13]. Acute CRS also involves RAAS and sympathetic activation, oxidative stress, inflammation, apoptosis, and mitochondrial injury, supporting the use of multi-biomarker panels rather than single-marker risk assessment [14].

Mineral metabolism provides another link between kidney dysfunction and cardiovascular injury. In CKD, FGF-23 rises as estimated GFR declines and phosphate handling becomes impaired. In adults with CKD stages 2–4, higher FGF-23 has been associated with greater left ventricular mass index and long-term adverse outcomes, including all-cause mortality, atrial fibrillation, and congestive HF. However, left ventricular hypertrophy (LVH) appears to explain only part of these associations, suggesting that FGF-23 may reflect broader CKD-related cardiovascular risk rather than a single myocardial hypertrophy pathway [15].

Inflammation and fibrosis markers further extend this profile. In CKD populations, higher growth differentiation factor-15 (GDF-15), galectin-3, and soluble ST2 have been independently associated with mortality, while GDF-15 has also been associated with HF events [16]. These data support a multi-biomarker model in which tubular injury, filtration impairment, inflammation, fibrosis, remodeling, and mineral metabolism disturbance jointly characterize cardiorenal risk.

**Figure 1:**
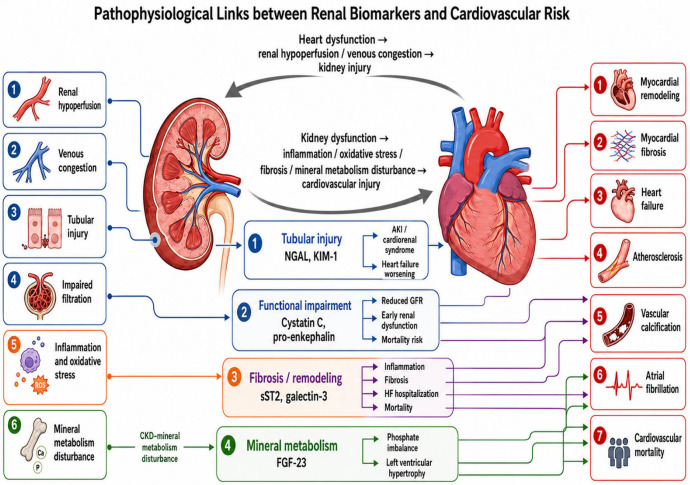
Pathophysiological links between renal biomarkers and cardiovascular risk. Cardiorenal dysfunction is driven by bidirectional interactions between the heart and kidneys. HF promotes renal hypoperfusion, venous congestion, neurohormonal activation, inflammation and tubular injury. In turn, kidney dysfunction contributes to endothelial dysfunction, oxidative stress, fibrosis, mineral metabolism disturbance, vascular calcification and myocardial remodeling. NGAL and KIM-1 reflect tubular injury; cystatin C and pro-enkephalin reflect functional renal impairment; ST2 and galectin-3 reflect inflammation, fibrosis and remodeling; and FGF-23 reflects mineral metabolism disturbance and CKD-related cardiovascular risk.

Clinically, the cardiorenal biomarker axis should therefore be interpreted in two complementary ways: first, according to the dominant biological pathway reflected by the biomarker, and second, according to the acute, subacute, or chronic phase of cardiorenal disease. NGAL and KIM-1 are most useful for identifying tubular injury, particularly in acute or unstable settings. Cystatin C and PENK refine functional renal assessment when creatinine is delayed or confounded. sST2 and galectin-3 mainly inform inflammatory, fibrotic, and remodeling pathways, while FGF-23 reflects the mineral metabolism axis that links CKD to vascular calcification, LVH, and long-term cardiovascular risk (Figure 1).

## 4. Tubular Injury Biomarkers: NGAL and KIM-1

### 4.1. NGAL

NGAL, also known as lipocalin-2, is a 25-kDa protein produced by several cell types, including neutrophils, renal tubular epithelial cells, macrophages, dendritic cells, cardiomyocytes, endothelial cells, and vascular smooth muscle cells. Its expression increases rapidly in response to ischemia, tubular injury, and inflammation, which explains its relevance as both a renal injury biomarker and an inflammatory mediator. In AKI, plasma NGAL may become detectable within approximately two hours and often rises earlier than SCr. Urinary NGAL mainly reflects tubular injury and increased tubular synthesis, whereas circulating NGAL may also be influenced by neutrophil activation, systemic inflammation, liver and lung production, and reduced renal clearance [17,18].

In HF, NGAL is relevant because tubular injury may occur before a measurable decline in glomerular filtration. Higher serum NGAL has been associated with increased mortality and composite adverse outcomes in patients with acute or chronic HF. Both serum and urinary NGAL have also been linked to WRF, supporting its interpretation as a marker of cardiorenal deterioration rather than a purely renal signal [17].

NGAL also has cardiovascular relevance beyond AKI detection. It is expressed in injured myocardium and atherosclerotic plaques, correlates with HF and coronary artery disease (CAD) severity, and may participate in inflammation, fibrosis, vascular remodeling, and matrix metalloproteinase-9 (MMP-9) stabilization [18,19]. In ST-elevation myocardial infarction, elevated admission NGAL has been independently associated with severe AKI or short-term mortality, indicating potential prognostic value in acute coronary settings [20]. NGAL should therefore be interpreted as an early tubular stress marker with broader links to systemic inflammation, acute coronary syndromes (ACS), HF progression, and adverse cardiovascular prognosis.

### 4.2. KIM-1

KIM-1, also known as T-cell immunoglobulin mucin-1, is a type 1 transmembrane glycoprotein with minimal expression in healthy kidneys. Its expression increases markedly after ischemic, toxic, or inflammatory tubular injury, mainly in damaged proximal tubular epithelial cells. The extracellular domain can be shed into urine, which makes urinary KIM-1 a relatively specific marker of proximal tubular damage rather than a simple indicator of reduced filtration [21,22].

KIM-1 has been studied in AKI, CKD, diabetic kidney disease, and immune-mediated renal injury. In AKI, it may rise before conventional functional markers and reflects tubular epithelial injury, dedifferentiation, repair, and phagocytosis of apoptotic or necrotic debris [21,22]. In CKD, persistent KIM-1 overexpression has been linked to tubular inflammation, macrophage recruitment, interstitial fibrosis, and faster kidney disease progression. In diabetic kidney disease, urinary KIM-1 has been associated with microalbuminuria, SCr, tubular damage, and early renal injury, although findings are less consistent in normoalbuminuric populations [22].

In cardiorenal settings, KIM-1 may help identify structural tubular injury when SCr changes are delayed, masked by congestion, or difficult to interpret during decongestive therapy. In HF, urinary KIM-1 has been associated with WRF, although its relationship with mortality and the combined outcome of mortality and HF hospitalization appears less consistent [17]. Higher plasma KIM-1 has also been independently associated with all-cause mortality in patients with hypertension and nondiabetic CKD, even after adjustment for cardiovascular risk factors, estimated glomerular filtration rate (eGFR), and albuminuria [23]. These observations support its potential value as a marker of tubular injury with broader prognostic relevance in selected high-risk renal and cardiovascular populations.

### 4.3. Cardiovascular Implications of Tubular Injury Biomarkers

NGAL and KIM-1 provide complementary information in cardiovascular patients because both reflect tubular stress or injury, but with different biological profiles. NGAL responds rapidly to tubular damage and systemic inflammation, whereas KIM-1 is more closely linked to proximal tubular epithelial injury. This distinction is clinically relevant in HF, where changes in SCr may reflect altered renal hemodynamics, congestion, or decongestive therapy rather than structural tubular damage.

In acute HF, higher urinary KIM-1 has been associated with death or HF readmission at 1 year, independently of conventional kidney and cardiac risk markers [24]. At the same time, creatinine-defined WRF does not always correspond to tubular injury. In patients with acute HF and mild AKI, urinary tubular injury biomarkers were not associated with worsening renal failure, dialysis, or short-term mortality, supporting the interpretation that some creatinine rises during acute decompensation are predominantly functional or hemodynamic [25].

Tubular biomarkers may also capture early kidney–vascular interactions before overt clinical disease. Urinary KIM-1 has been associated with coronary stenosis and coronary artery calcium after adjustment for established cardiovascular risk factors [26]. In chronic HF, urinary NGAL and KIM-1 appear to be higher in heart failure with preserved ejection fraction (HFpEF) than in heart failure with reduced ejection fraction (HFrEF), particularly when eGFR is preserved, suggesting that tubular dysfunction may be present despite apparently stable glomerular markers [27]. After cardiac valve replacement, postoperative increases in KIM-1 and NGAL have been observed in patients developing AKI, consistent with their potential value as early postoperative renal injury markers [28].

NGAL and KIM-1 may therefore help separate subclinical tubular injury from isolated functional renal impairment and refine cardiorenal phenotyping in selected high-risk cardiovascular settings. Other tubular or tubular-stress biomarkers, including liver-type fatty acid-binding protein (L-FABP), interleukin-18 (IL-18), tissue inhibitor of metalloproteinases-2 × insulin-like growth factor-binding protein 7 (TIMP-2 × IGFBP7), monocyte chemoattractant protein-1 (MCP-1), and C–C motif chemokine ligand 14 (CCL14), have also shown potential in acute HF and AKI. Their integration into cardiovascular risk stratification remains less developed than that of NGAL and KIM-1, and routine clinical use will require stronger validation [24,25].

The main characteristics, clinical relevance, and limitations of NGAL and KIM-1 as tubular injury biomarkers in cardiorenal disease are summarized in Table 3.

## 5. Functional Renal Biomarkers Beyond Creatinine: Cystatin C and Pro-Enkephalin

### 5.1. Cystatin C

Cystatin C is a low-molecular-weight cysteine protease inhibitor produced at a relatively constant rate by all nucleated cells. Compared with creatinine, which is influenced by muscle mass, diet, age, sex, and tubular secretion, cystatin C is freely filtered by the glomerulus, then reabsorbed and catabolized by proximal tubular cells. These properties make it a useful endogenous marker for refining the assessment of kidney function [29]. Its main clinical value lies in situations where creatinine-based estimated glomerular filtration rate (eGFRcr) may be misleading, such as in older adults, frail patients, individuals with reduced muscle mass, or those with non-standard body composition. Equations combining creatinine and cystatin C generally provide more accurate GFR estimation than either marker alone, and cystatin C may help confirm CKD when creatinine-based eGFR values are borderline or clinically uncertain [29].

Cystatin C also provides prognostic information beyond creatinine-based assessment. Discordance between cystatin C-based eGFR and creatinine-based eGFR is clinically relevant, particularly when eGFRcys is substantially lower than eGFRcr. This pattern has been associated with higher risks of AKI, kidney failure requiring replacement therapy, atherosclerotic cardiovascular disease (ASCVD), HF, and all-cause mortality [30]. Cystatin C-based definitions of CKD also appear to show stronger associations with cardiovascular mortality than creatinine-based definitions in population-based settings [31].

The relevance of cystatin C extends to atherosclerotic disease. Higher serum cystatin C has been associated with greater coronary atherosclerotic plaque burden, and genetic analyses have supported a possible causal relationship with coronary atherosclerosis [32]. Large-scale population data also indicate that creatinine- and cystatin C-based eGFR may capture different cardiovascular risk patterns, suggesting that cystatin C can add information not fully reflected by creatinine alone [33].

### 5.2. Pro-Enkephalin

PENK 119–159, also referred to as penKid, is a stable fragment derived from the proenkephalin precursor and is used as a surrogate marker of the endogenous enkephalin system. Biologically active enkephalins are rapidly degraded and have limited analytical stability, whereas PENK has a longer in vitro half-life and can be measured reliably in plasma. Because it is mainly eliminated by glomerular filtration and shows a close inverse relationship with kidney function, PENK has been proposed as a functional biomarker that may reflect dynamic changes in renal impairment [34].

In acute HF, PENK may be useful because renal deterioration can develop early and may not be detected promptly by creatinine. Higher PENK measured at presentation has been independently associated with WRF within 48 h and with short- and long-term mortality outcomes [35]. This supports its potential value in acute cardiorenal assessment, although its interpretation still depends on clinical context and assay availability.

PENK has also shown relevance in broader AKI settings. Across observational data, PENK has demonstrated moderate diagnostic performance for AKI detection, with reported cut-off values varying according to clinical setting and population [36]. In ACS, higher PENK has been associated with in-hospital AKI and 30-day mortality, and PENK-based risk models have improved cardiorenal risk prediction [37]. Compared with cystatin C, however, PENK remains less widely available and less established in routine practice. Its main current value lies in acute-care research and selected high-risk settings, where earlier recognition of renal functional deterioration may refine cardiorenal risk assessment.

The main analytical advantages, cardiovascular relevance, and clinical limitations of cystatin C and pro-enkephalin as functional renal biomarkers beyond creatinine are summarized in Table 4.

## 6. Biomarkers of Fibrosis and Remodeling: ST2 and Galectin-3

### 6.1. sST2

Soluble suppression of tumorigenicity-2 (sST2) is a circulating member of the interleukin-1 receptor family and reflects myocardial stress, inflammation, and fibrosis. The sST2 pathway includes two main forms: the transmembrane receptor sST2L and the soluble receptor sST2. Under physiological stress, interleukin-33 binding to sST2L exerts protective cardiovascular effects by limiting cardiomyocyte hypertrophy, apoptosis, and fibrosis. By contrast, sST2 acts as a decoy receptor for interleukin-33, reducing sST2L-mediated signaling and favoring myocardial fibrosis and ventricular remodeling [38,39]. Although sST2 is released in response to myocardial injury and mechanical stress, it is not exclusively cardiac in origin; endothelial cells, immune cells, and extracardiac tissues may also contribute to circulating concentrations [38,39].

In HF, sST2 is used mainly as a prognostic rather than diagnostic biomarker. Compared with natriuretic peptides, sST2 appears less influenced by age, sex, body mass index, atrial fibrillation, anemia, HF etiology, and kidney function, and it shows lower intra-individual variation [38]. This is relevant in cardiorenal populations, where the interpretation of natriuretic peptides may be complicated by renal dysfunction. Higher sST2 concentrations have been associated with all-cause mortality, cardiovascular mortality, HF hospitalization, and improved risk reclassification when added to conventional prognostic models [39]. Serial sST2 measurements may also provide information on dynamic remodeling risk and treatment response [39].

The value of sST2 in cardiorenal disease is supported by data from patients with coronary disease, HF, and renal impairment. Higher sST2 has been associated with incident HF, atrial fibrillation, and death, and its prognostic value appears to be less affected by renal function than that of some conventional cardiac biomarkers [40]. In ADHF with renal insufficiency, sST2 concentrations are less strongly influenced by the degree of renal dysfunction than B-type natriuretic peptide (BNP), while predischarge sST2 has been associated with death or HF readmission [41]. These observations support the interpretation of sST2 as a fibrosis- and remodeling-related biomarker with potential value when renal dysfunction limits the clarity of conventional cardiac biomarker assessment.

### 6.2. Galectin-3

Galectin-3 is a β-galactoside-binding lectin and the only chimera-type member of the galectin family. It is expressed in several tissues and cell types, including macrophages, myeloid cells, kidney, heart, and vascular tissue. Galectin-3 participates in cell–cell and cell–matrix interactions, macrophage activation, inflammation, apoptosis, angiogenesis, and fibrogenesis [42,43]. In CVD, its relevance is mainly linked to inflammatory amplification, fibroblast activation, collagen deposition, and extracellular matrix remodeling, making it a marker of active fibrotic biology rather than a passive indicator of myocardial fibrosis [43].

In HF, galectin-3 has been associated with myocardial remodeling, fibrosis, ventricular dysfunction, and adverse prognosis. Higher circulating concentrations have been linked to mortality in both general and HF populations, and galectin-3 has been evaluated as an additive risk-stratification biomarker in HF [43]. Its clinical interpretation, however, is less straightforward than that of natriuretic peptides. Circulating galectin-3 may reflect extracardiac sources, particularly renal dysfunction, which is directly relevant in cardiorenal populations [42].

Renal data further support galectin-3 as a cardiorenal remodeling biomarker. In AKI and CKD, galectin-3 is involved in inflammatory amplification and renal fibrogenesis; circulating concentrations are elevated in both settings, and higher levels have been associated with CKD progression [44]. In symptomatic HFrEF, galectin-3 has been associated with the development of CRS and has shown moderate discriminatory performance for this complication [45]. Thus, galectin-3 should be interpreted as a marker of inflammation-driven fibrosis and remodeling across both cardiac and renal compartments. sST2 and galectin-3 therefore provide complementary information within the fibrosis and remodeling domain. sST2 is more closely linked to myocardial stress and IL-33/ST2 signaling, whereas galectin-3 reflects macrophage activation, extracellular matrix remodeling, and fibrogenesis across several tissues. In cardiorenal disease, neither biomarker should be viewed as purely cardiac. Their value lies in identifying inflammatory and fibrotic pathways that may contribute to HF progression, renal deterioration, and adverse cardiovascular outcomes.

## 7. FGF-23 and the Mineral Metabolism–Cardiovascular Axis

FGF-23 is a bone-derived phosphaturic hormone produced mainly by osteocytes and osteoblasts. It regulates phosphate and vitamin D metabolism through the FGF receptor–Klotho axis. In the kidney, FGF-23 downregulates sodium–phosphate cotransporters in proximal tubules, increases phosphaturia, suppresses 1,25-dihydroxyvitamin D synthesis, and modulates parathyroid hormone secretion [46,47]. In CKD, FGF-23 is one of the earliest detectable abnormalities of mineral metabolism. Its concentration rises progressively as GFR declines, initially as an adaptive response to maintain phosphate balance. With advancing CKD, reduced nephron mass, Klotho deficiency, and phosphate retention contribute to FGF-23 resistance and to the broader phenotype of CKD-mineral and bone disorder (CKD-MBD) [46,47].

The cardiovascular relevance of FGF-23 lies in its connection with myocardial and vascular injury. Elevated FGF-23 has been associated with LVH, cardiac fibrosis, arterial stiffness, atrial fibrillation, atherosclerosis, and higher cardiovascular and all-cause mortality [48]. Mechanistic data indicate that FGF-23 may promote cardiomyocyte hypertrophy through FGFR4/calcineurin/NFAT signaling, independently of Klotho. It may also interact with inflammation, RAAS activation, and sodium handling, although the relative contribution of these pathways in clinical disease remains difficult to separate [46,48].

FGF-23 is also linked to the vascular phenotype of CKD. Higher FGF-23 has been positively associated with arterial calcification, carotid intima–media thickness, and pulse wave velocity, whereas Klotho shows inverse associations with calcification and arterial thickness [49]. CKD-related vascular calcification is further promoted by hyperphosphatemia, hypercalcemia, oxidative stress, inflammation, and loss of calcification inhibitors. These processes favor osteogenic transformation of vascular smooth muscle cells and contribute to arterial stiffness [50]. FGF-23 therefore differs from tubular injury, filtration, and fibrosis biomarkers because it reflects a mineral metabolism pathway through which kidney dysfunction may contribute to vascular calcification, myocardial hypertrophy, and cardiovascular risk.

The fibrosis-, remodeling-, and mineral metabolism-related biomarkers that complement tubular and functional renal markers are summarized in Table 5.

## 8. Clinical Applications: Toward Biomarker-Guided Cardiovascular Risk Stratification

Clinical application of renal and cardiorenal biomarkers should start from a pragmatic premise: creatinine, eGFR, albuminuria, natriuretic peptides, and troponins remain necessary, but they do not fully characterize cardiorenal risk. In CRS, conventional markers may be delayed, nonspecific, or difficult to interpret because renal dysfunction, congestion, inflammation, and treatment-related hemodynamic changes often coexist. Biomarkers are therefore most useful when interpreted in relation to specific clinical questions, such as whether renal worsening during decongestion reflects structural tubular injury or functional hemodynamic change, whether persistent congestion is contributing to renal impairment, or whether a patient is at imminent risk of AKI [51].

A biomarker-guided approach may be particularly relevant in high-risk cardiovascular populations, including patients with HF, ACS, CKD, diabetes, hypertension, and AKI. In acute CRS, laboratory panels may be more informative than isolated markers. In patients with acute HF at risk of type 1 CRS, cystatin C, changes in SCr, KIM-1, NGAL, and TIMP-2 × IGFBP7 may support earlier identification of AKI. Conversely, in patients with AKI at risk of acute HF or type 3 CRS, high-sensitivity troponin, BNP, and N-terminal pro-B-type natriuretic peptide (NT-proBNP) may help detect cardiac involvement [52].

The rationale for this approach reflects the biology of kidney injury. SCr and urine output are functional markers and may miss subclinical AKI, in which tubular epithelial injury precedes measurable decline in glomerular filtration. Newer biomarkers capture different injury domains: cystatin C and PENK reflect filtration changes; NGAL, KIM-1, N-acetyl-β-D-glucosaminidase (NAG), and L-FABP reflect tubular damage; TIMP-2 and IGFBP7 reflect tubular stress and cell-cycle arrest; interleukin-18 (IL-18) and interleukin-9 (IL-9) reflect intrarenal inflammation; and CCL14 may identify persistent severe AKI [53].

In practice, these markers could complement conventional assessment by helping assign patients to a dominant cardiorenal phenotype, such as tubular injury, filtration impairment, congestion, myocardial injury, fibrosis/remodeling, inflammation, or mineral metabolism disturbance. Such phenotyping may support earlier renal injury detection, prediction of CKD progression, identification of high-risk HF patients, assessment of CRS, cardiovascular risk stratification, and monitoring of treatment response. Interpretation must remain contextual. Temporary creatinine increases during decongestion do not always indicate structural kidney injury, while troponin and NT-proBNP may be chronically elevated in renal dysfunction, even outside ACS or acute HF [54].

Beyond early diagnosis and prognosis, the reviewed biomarkers may also have indirect relevance for clinical management. However, this should be interpreted cautiously, because most available evidence supports biomarker use for risk stratification, phenotype recognition, and monitoring rather than for definitive biomarker-guided therapeutic decisions. These interpretations may help clinicians decide which patients require closer follow-up, repeated renal and cardiac biomarker assessment, more careful evaluation of congestion and renal function, or earlier multidisciplinary cardiorenal assessment (Table 6).

In CKD, multi-biomarker strategies may improve cardiovascular risk prediction by integrating signals from myocardial stress, tubular injury, inflammation, extracellular matrix remodeling, and vascular calcification. A protein panel combining NT-proBNP, KIM-1, osteopontin, and TIMP-1 has been shown to predict incident cardiovascular events and to identify higher-risk patients across CKD stages [55]. This supports the concept that panels reflecting several biological pathways may be more informative than isolated renal or cardiac markers when selecting patients who may require closer monitoring or intensified prevention.

In ACS, biomarker profiling illustrates the overlap between kidney dysfunction and cardiovascular risk. Inflammatory, angiogenic, and kidney-related biomarkers tend to increase as kidney function declines. Among these markers, endothelial cell-specific molecule-1, FGF-23, and KIM-1 have been associated with major adverse cardiovascular events and death, while FGF-23 appears to retain independent prognostic value after adjustment for other biomarker signals [56]. This finding is consistent with the relevance of mineral metabolism and inflammatory–vascular pathways in ACS, including in patients with impaired kidney function.

Post-AKI follow-up is another setting in which biomarker-guided risk stratification may be clinically useful. Vascular biomarker panels measured after hospitalization can identify phenotypes related to vascular injury, repair, and lower-risk profiles. Patients with a vascular injury phenotype appear to have higher subsequent HF risk, and biomarker data may improve prediction of HF or death beyond clinical variables alone [57]. These findings support the value of post-discharge reassessment, especially in patients whose renal function appears to recover but who remain at increased cardiovascular risk.

Biomarker-guided strategies should also be integrated into the broader cardiovascular–kidney–metabolic framework. Albuminuria remains central for screening and monitoring kidney and cardiovascular risk in diabetes, hypertension, and CVD, while NT-proBNP and high-sensitivity troponin support HF detection, ACS diagnosis, and long-term cardiovascular risk stratification. These markers require careful interpretation: albuminuria is biologically variable, NT-proBNP is influenced by age, CKD, atrial fibrillation, and obesity, and troponin may reflect chronic myocardial injury rather than acute plaque-related ischemia [58]. A practical workflow would therefore begin with identification of a high-risk patient, followed by conventional assessment, targeted biomarker testing, dominant phenotype assignment, risk categorization, and individualized monitoring or treatment intensity.

**Figure 2:**
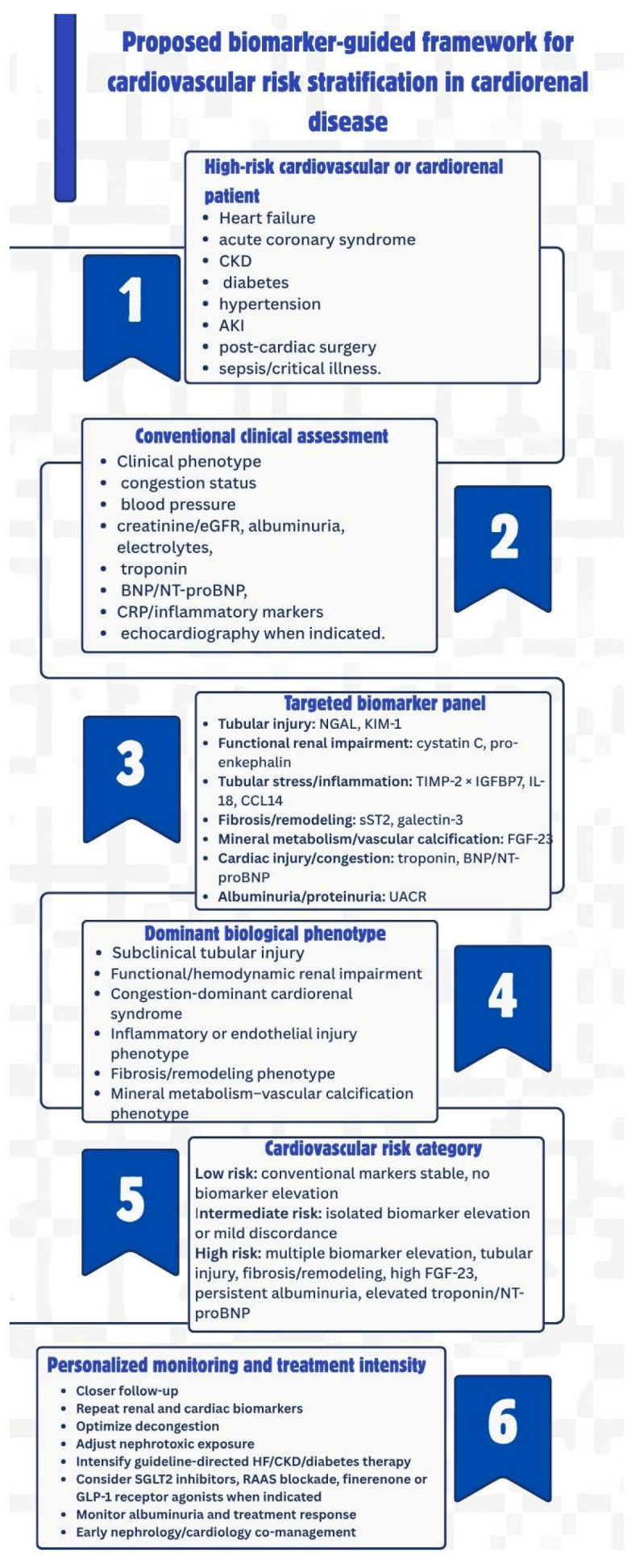
Proposed biomarker-guided framework for cardiovascular risk stratification. Abbreviations: ACS, acute coronary syndrome; AKI, acute kidney injury; BNP, B-type natriuretic peptide; CCL14, C–C motif chemokine ligand 14; CKD, chronic kidney disease; CRP, C-reactive protein; eGFR, estimated glomerular filtration rate; FGF-23, fibroblast growth factor-23; GLP-1 RA, glucagon-like peptide-1 receptor agonist; HF, heart failure; IGFBP7, insulin-like growth factor-binding protein 7; IL-18, interleukin-18; KIM-1, kidney injury molecule-1; NGAL, neutrophil gelatinase-associated lipocalin; NT-proBNP, N-terminal pro-B-type natriuretic peptide; PENK, pro-enkephalin; RAAS, renin–angiotensin–aldosterone system; SGLT2, sodium–glucose cotransporter-2; sST2, soluble suppression of tumorigenicity-2; TIMP-2, tissue inhibitor of metalloproteinases-2; UACR, urinary albumin-to-creatinine ratio.

This proposed biomarker-guided framework for cardiovascular risk stratification is illustrated in Figure 2, integrating conventional assessment with targeted biomarker panels to identify dominant cardiorenal phenotypes and guide individualized monitoring and treatment intensity.

## 9. Current Limitations and Future Directions

Renal and cardiorenal biomarkers have strong biological rationale and increasing clinical support, but they are not yet ready for universal routine implementation. One limitation is that many clinical definitions still depend on SCr, eGFR, and urine output, although these markers are delayed, functional, and nonspecific. In AKI, creatinine may remain unchanged during early tubular injury and may rise only after substantial functional decline. Urine output is also difficult to interpret, as it can be influenced by hemodynamics, diuretic exposure, and transient physiological responses [5,59]. This creates a diagnostic gap between structural kidney injury and conventional criteria, particularly in cardiovascular patients exposed to congestion, hypoperfusion, contrast agents, nephrotoxic drugs, or critical illness.

Novel biomarkers have their own limitations. NGAL, KIM-1, L-FABP, TIMP-2 × IGFBP7, cystatin C, PENK, and inflammatory markers may improve early detection and phenotyping, but their diagnostic performance varies according to clinical setting, assay platform, sampling time, biological matrix, and patient population [59]. Several non-renal factors can also influence biomarker concentrations, including inflammation, sepsis, diabetes, obesity, malignancy, corticosteroid exposure, thyroid dysfunction, muscle mass, proteinuria, and CKD stage. These influences reduce specificity and complicate interpretation. For many biomarkers, accepted cut-off values are still lacking, and assay standardization remains incomplete [5,59,60]. Cost and availability also add barriers, especially for multi-marker platforms, omics-based diagnostics, and point-of-care tools.

Clinical actionability remains another major challenge. A biomarker may improve diagnosis or prognosis without proving that biomarker-guided management improves patient outcomes. For routine adoption, biomarkers must show that they can change clinical decisions, improve patient-centered outcomes, and remain cost-effective in prospective studies. At present, the strongest evidence supports their use for risk stratification and phenotyping, while outcome-driven interventional data remain limited [5,60].

Future research should focus on prospective validation cohorts, harmonized assays, pre-analytical standardization, and clinically meaningful thresholds adjusted for age, sex, kidney function, and comorbidity burden. Multi-biomarker panels should be incorporated into risk models that combine clinical variables, cardiac and renal biomarkers, imaging findings, and phenotype-based assessment. Multi-omics approaches, including genomics, proteomics, metabolomics, and transcriptomics, may improve disease classification and support more precise cardiorenal phenotyping. Artificial intelligence may also help integrate complex biomarker and clinical data for real-time risk prediction and individualized treatment selection [60,61]. However, AI-based cardiovascular prediction models still require external validation, transparency, reproducibility, and bias assessment before clinical deployment. Many published models remain at high risk of bias and lack independent external validation [61]. The next step is therefore not simply to identify additional biomarkers, but to translate biologically meaningful signals into standardized, interpretable, and clinically useful pathways.

## 10. Conclusions

Emerging renal and cardiorenal biomarkers may refine cardiovascular risk assessment beyond SCr and creatinine-based eGFR. Conventional renal markers remain necessary in routine practice, but they often reflect functional decline after injury has already developed and provide limited information on pathway-specific cardiorenal damage. In this context, NGAL and KIM-1 may help identify tubular stress and proximal tubular injury before overt functional deterioration becomes apparent. Cystatin C and PENK add information on renal functional impairment, particularly when creatinine is influenced by muscle mass, frailty, or acute hemodynamic changes. sST2 and galectin-3 reflect inflammation, fibrosis, and cardiac or renal remodeling, while FGF-23 captures the mineral metabolism pathway linking CKD to vascular calcification, LVH, arterial stiffness, and cardiovascular risk.

These biomarkers support a more integrated view of cardiorenal disease, in which tubular injury, altered filtration, congestion, inflammation, fibrosis, and mineral metabolism disturbance are assessed as related biological domains rather than isolated abnormalities. Their most plausible clinical value lies in earlier injury detection, cardiorenal phenotyping, identification of high-risk cardiovascular patients, and individualized monitoring. Routine implementation, however, remains limited by assay variability, uncertain thresholds, biological confounding, cost, and the lack of definitive evidence that biomarker-guided management improves clinical outcomes. Before these tools can be incorporated more broadly into clinical decision-making, prospective validation, assay standardization, and clinically actionable multi-biomarker risk models are needed.

## Figures and Tables

**Table 1:** Classification of cardiorenal syndrome according to the primary organ injury and time course.

CRS Type	Primary Disorder	Secondary Dysfunction	Time Course and Clinical Meaning
Type 1 CRS	Acute cardiac dysfunction, such as acute decompensated HF or acute coronary syndrome	AKI	Acute cardiac injury leads to acute renal dysfunction, often through renal hypoperfusion, venous congestion, inflammation, and neurohormonal activation.
Type 2 CRS	Chronic cardiac dysfunction, especially chronic heart failure	Progressive CKD	Chronic cardiac disease contributes to sustained renal impairment through persistent congestion, reduced renal perfusion, RAAS activation, and inflammatory or fibrotic pathways.
Type 3 CRS	Acute kidney injury	Acute cardiac dysfunction, such as heart failure, arrhythmia, ischemia, or myocardial injury	Acute renal injury promotes cardiac complications through volume overload, electrolyte imbalance, uremic toxins, inflammation, and oxidative stress.
Type 4 CRS	CKD	Cardiovascular disease, including left ventricular hypertrophy, heart failure, vascular calcification, and atherosclerosis	Chronic renal dysfunction promotes long-term cardiovascular injury through anemia, CKD-MBD, inflammation, endothelial dysfunction, oxidative stress, and vascular remodeling.
Type 5 CRS	Systemic disorders, such as sepsis, diabetes, inflammatory disease, or critical illness	Simultaneous cardiac and renal dysfunction	A systemic condition causes parallel injury to both organs through inflammation, microvascular dysfunction, metabolic stress, and hemodynamic instability.

Abbreviations: AKI, acute kidney injury; CKD, chronic kidney disease; CKD-MBD, chronic kidney disease–mineral and bone disorder; CRS, cardiorenal syndrome; HF, heart failure; RAAS, renin–angiotensin–aldosterone system.

**Table 2:** Acute and chronic cardiorenal biomarker profiles according to dominant pathophysiological phase.

Clinical Phase	Dominant Pathophysiological Process	Main Biomarkers	Clinical Interpretation
Acute phase	Tubular stress, acute tubular injury, acute functional renal deterioration, hemodynamic instability, acute HF, ACS, post-cardiac surgery AKI	NGAL, KIM-1, cystatin C, PENK, TIMP-2 × IGFBP7, IL-18, CCL14	May support earlier recognition of AKI or acute cardiorenal deterioration before creatinine-based criteria become evident; useful when renal worsening may reflect either structural tubular injury or functional hemodynamic change.
Subacute or transition phase	Incomplete recovery after AKI, persistent congestion, residual tubular injury, post-discharge cardiorenal risk	cystatin C, PENK, NGAL, KIM-1, albuminuria/proteinuria, selected vascular or inflammatory biomarkers	May help identify patients who appear clinically stable but remain at increased risk of CKD progression, HF events, rehospitalization, or cardiovascular mortality after an acute episode.
Chronic phase	CKD, chronic HF, fibrosis, myocardial and renal remodeling, endothelial dysfunction, CKD-MBD, vascular calcification, long-term cardiovascular injury	cystatin C, sST2, galectin-3, FGF-23, KIM-1, albuminuria, GDF-15	May refine long-term risk stratification by identifying persistent filtration impairment, fibrotic remodeling, mineral metabolism disturbance, vascular injury, and higher risk of HF, LVH, atrial fibrillation, atherosclerosis, and mortality.

Abbreviations: ACS, acute coronary syndrome; AKI, acute kidney injury; CCL14, C–C motif chemokine ligand 14; CKD, chronic kidney disease; CKD-MBD, chronic kidney disease–mineral and bone disorder; FGF-23, fibroblast growth factor-23; GDF-15, growth differentiation factor-15; HF, heart failure; IGFBP7, insulin-like growth factor-binding protein 7; IL-18, interleukin-18; KIM-1, kidney injury molecule-1; LVH, left ventricular hypertrophy; NGAL, neutrophil gelatinase-associated lipocalin; PENK, pro-enkephalin; sST2, soluble suppression of tumorigenicity-2; TIMP-2, tissue inhibitor of metalloproteinases-2.

**Table 3:** Tubular injury biomarkers with cardiovascular relevance.

Biomarker	Main Source	Biological Meaning	Renal Relevance	Cardiovascular Relevance	Main Limitations
NGAL	Neutrophils, renal tubular epithelial cells, cardiomyocytes, endothelial cells, and vascular smooth muscle cells [17,18,19].	Rapid tubular stress/injury response; also reflects inflammation and MMP-9-related vascular remodeling [17,18,19].	May rise earlier than serum creatinine SCr in AKI. Urinary NGAL mainly reflects tubular synthesis and injury, while circulating NGAL is more influenced by systemic inflammation and reduced renal clearance [17,18].	Associated with WRF in HF, adverse outcomes in acute or chronic HF, and severe AKI or 30-day mortality in STEMI [15,20].	Limited kidney specificity; affected by systemic inflammation, infection, sepsis, obesity, diabetes, malignancy, extracardiac production, and reduced renal clearance [17,18,25].
KIM-1	Injured proximal tubular epithelial cells, with urinary shedding after tubular injury [21,22].	Proximal tubular epithelial injury, repair response, and chronic tubulointerstitial inflammation/fibrosis [21,22].	Marker of structural tubular injury in AKI, CKD progression, diabetic kidney disease, and tubulointerstitial damage [21,22].	Associated with WRF in HF, death or HF readmission in acute HF, all-cause mortality in high-risk CKD, and coronary atherosclerosis markers [17,23,24,26].	Less established in routine practice than NGAL; optimal thresholds, sampling time, urine concentration correction, assay standardization, and clinical decision pathways remain unresolved [21,22,23,24].

Abbreviations: AKI, acute kidney injury; CKD, chronic kidney disease; HF, heart failure; KIM-1, kidney injury molecule-1; MMP-9, matrix metalloproteinase-9; NGAL, WRF, worsening renal function; neutrophil gelatinase-associated lipocalin; STEMI, ST-elevation myocardial infarction.

**Table 4:** Functional renal biomarkers beyond creatinine.

Biomarker	Advantage over Creatinine	Main Clinical Use	Cardiovascular Association	Key Limitations
Cystatin C	Less dependent on muscle mass, diet, and tubular secretion; improves GFR estimation when combined with creatinine [29].	Refinement of eGFR; confirmation of CKD when creatinine-based eGFR is borderline or uncertain; useful in older, frail, or low-muscle-mass patients [29,30].	Lower eGFRcys relative to eGFRcr has been associated with higher risk of AKI, kidney failure, ASCVD, HF, and death. Cystatin C-based CKD shows stronger associations with cardiovascular mortality than creatinine-based CKD in population-based settings [30,31]. Higher cystatin C has also been associated with coronary plaque burden [32].	Less available and more expensive than creatinine; affected by inflammation, obesity, smoking, thyroid dysfunction, glucocorticoids, and some comorbid conditions. Discordant eGFRcr/eGFRcys values require clinical interpretation [29,30,33].
Pro-enkephalin/PENK 119–159	Stable plasma peptide mainly eliminated by glomerular filtration; may reflect dynamic renal functional change earlier than creatinine in acute settings [34,36].	Emerging marker for AKI, WRF, and cardiorenal deterioration, especially in acute HF, sepsis, critical illness, and ACS [34,35,36,37].	Higher PENK has been associated with WRF and mortality in acute HF, and with in-hospital AKI and 30-day mortality in ACS [35,37].	Less widely available than cystatin C; limited routine implementation; evidence remains mainly observational; optimal cut-offs vary across clinical settings, assays, and comorbidity profiles [34,35,36,37].

Abbreviations: AKI, acute kidney injury; ASCVD, atherosclerotic cardiovascular disease; CKD, chronic kidney disease; eGFR, estimated glomerular filtration rate; eGFRcr, creatinine-based estimated glomerular filtration rate; eGFRcys, cystatin C-based estimated glomerular filtration rate; CVD, Cardiovascular disease; GFR, glomerular filtration rate; HF, heart failure; WRF, worsening renal function; ACS, acute coronary syndromes; PENK, pro-enkephalin.

**Table 5:** Biomarkers of fibrosis, remodeling and mineral metabolism.

Biomarker	Main Pathway	Renal Relevance	Cardiovascular Relevance	Potential Clinical Role
sST2	IL-33/ST2 axis; myocardial stretch, inflammation, fibrosis, and ventricular remodeling. sST2 acts as a decoy receptor that attenuates IL-33/ST2L protective signaling [38,39].	Less influenced by renal function than natriuretic peptides; may remain interpretable when renal dysfunction complicates BNP or NT-proBNP assessment [38,41].	Associated with adverse outcomes in acute and chronic HF, including HF hospitalization and mortality. Prognostic value appears relatively preserved in renal insufficiency [39,40,41].	Risk stratification in HF and coronary disease, especially when renal dysfunction limits interpretation of conventional cardiac biomarkers [38,39,40,41].
Galectin-3	Macrophage activation, inflammation, fibroblast activation, collagen deposition, extracellular matrix remodeling, and fibrosis [42,43].	Involved in AKI, CKD progression, and renal fibrogenesis. Higher circulating concentrations have been associated with CKD progression [44].	Associated with myocardial fibrosis, ventricular remodeling, HF prognosis, and CRS development [43,45].	Additive risk stratification in HF and CRS; marker of inflammation-driven myocardial and renal fibrosis [42,43,44].
FGF-23	Phosphate and vitamin D metabolism; CKD-MBD; FGF receptor–Klotho signaling; vascular calcification and myocardial hypertrophy pathways [46,47].	Rises early as GFR declines and reflects phosphate imbalance, Klotho deficiency, CKD-MBD, and secondary hyperparathyroidism [46,47].	Associated with LVH, cardiac fibrosis, arterial stiffness, atrial fibrillation, atherosclerosis, and cardiovascular mortality. Higher FGF-23 is also linked to arterial calcification, carotid intima–media thickness, and pulse wave velocity [48,49].	Risk stratification in CKD-related CVD, particularly when mineral metabolism disturbance may contribute to vascular calcification, arterial stiffness, and LVH [46,47,48,49,50].

Abbreviations: AKI, acute kidney injury; BNP, B-type natriuretic peptide; CKD, chronic kidney disease; CKD-MBD, chronic kidney disease–mineral and bone disorder; FGF-23, fibroblast growth factor-23; GFR, glomerular filtration rate; HF, heart failure; IL, interleukin; NT-proBNP, N-terminal pro-B-type natriuretic peptide; sST2, soluble suppression of tumorigenicity-2; CRS, cardiorenal syndrome; LVH, left ventricular hypertrophy.

**Table 6:** Potential management relevance of biomarker profiles.

Biomarker Profile	Main Biological Meaning Supported by the Reviewed Literature	Possible Management Relevance
NGAL and/or KIM-1 elevation	Tubular stress or structural tubular injury, particularly in AKI, acute HF, ACS, post-cardiac surgery, or other high-risk settings	May identify patients in whom renal dysfunction deserves closer monitoring and careful clinical interpretation, especially when creatinine changes may be delayed, functional, or hemodynamically driven.
Cystatin C and/or PENK elevation	Functional renal impairment beyond creatinine-based assessment	May support more accurate risk stratification and closer reassessment of renal function when creatinine is affected by muscle mass, frailty, acute hemodynamic change, or delayed kinetics.
sST2 and/or galectin-3 elevation	Inflammation, fibrosis, myocardial or renal remodeling, and higher HF-related risk	May identify patients who require closer longitudinal follow-up for HF progression, remodeling burden, or cardiorenal deterioration.
FGF-23 elevation	Mineral metabolism disturbance, CKD-MBD biology, vascular calcification risk, LVH, and long-term cardiovascular risk	May support closer evaluation of CKD-related cardiovascular risk and mineral metabolism status within standard nephrology and cardiovascular assessment.
Multiple elevated biomarkers across domains	Combined tubular injury, functional impairment, inflammation, remodeling, and/or mineral metabolism disturbance	May suggest a high-risk cardiorenal phenotype in which closer follow-up and multidisciplinary assessment may be clinically appropriate.

Abbreviations: ACS, acute coronary syndrome; AKI, acute kidney injury; CKD-MBD, chronic kidney disease–mineral and bone disorder; FGF-23, fibroblast growth factor-23; HF, heart failure; KIM-1, kidney injury molecule-1; LVH, left ventricular hypertrophy; NGAL, neutrophil gelatinase-associated lipocalin; PENK, pro-enkephalin; sST2, soluble suppression of tumorigenicity-2.

## Data Availability

No new data were created or analyzed in this study. Data sharing is not applicable to this article.

## Abbreviations

The following abbreviations are used in this manuscript:

ACS	Acute coronary syndrome
ADHF	Acute decompensated heart failure
AKI	Acute kidney injury
ASCVD	Atherosclerotic cardiovascular disease
BNP	B-type natriuretic peptide
CAD	Coronary artery disease
CCL14	C–C motif chemokine ligand 14
CKD	Chronic kidney disease
CRS	Cardiorenal syndrome
CVD	Cardiovascular disease
eGFR	Estimated glomerular filtration rate
eGFRcr	Creatinine-based estimated glomerular filtration rate
FGF-23	Fibroblast growth factor-23
GDF-15	Growth differentiation factor-15
GFR	Glomerular filtration rate
HF	Heart failure
HFpEF	Heart failure with preserved ejection fraction
HFrEF	Heart failure with reduced ejection fraction
IL	Interleukin
KIM-1	Kidney injury molecule-1
L-FABP	Liver-type fatty acid-binding protein
LVH	Left ventricular hypertrophy
MCP-1	Monocyte chemoattractant protein-1
MMP-9	Matrix metalloproteinase-9
NAG	N-acetyl-β-D-glucosaminidase
NGAL	Neutrophil gelatinase-associated lipocalin
NT-proBNP	N-terminal pro-B-type natriuretic peptide
PENK	Pro-enkephalin
RAAS	Renin–angiotensin–aldosterone system
SCr	Serum creatinine
sST2	Soluble suppression of tumorigenicity-2
STEMI	ST-elevation myocardial infarction
WRF	Worsening renal function
